# Nanovesicles as Vanillin Carriers for Antimicrobial Applications

**DOI:** 10.3390/membranes13010095

**Published:** 2023-01-11

**Authors:** Verdiana Marchianò, Maria Matos, Miriam López, Shihan Weng, Esther Serrano-Pertierra, Susana Luque, M. Carmen Blanco-López, Gemma Gutiérrez

**Affiliations:** 1 Department of Chemical and Environmental Engineering, University of Oviedo, Julián Clavería 8, 33006 Oviedo, Spain; 2 Department of Physical and Analytical Chemistry, University of Oviedo, Julián Clavería 8, 33006 Oviedo, Spain; 3 Instituto Universitario de Biotecnología de Asturias, University of Oviedo, 33006 Oviedo, Spain

**Keywords:** vesicles, vanillin, encapsulation, lyophilization, gelatin films, antimicrobial properties

## Abstract

Vanillin is a natural compound easily extracted from plants. It has neuroprotective, anti-carcinogenic, antioxidant, antimicrobial, and anti-biofilm properties. It also presents high volatility, high hydrophilicity, and low bioavailability. Nanomaterials can be used to improve pharmacodynamics, solubility, and stability and to enhance pharmacokinetics. In this work, non-ionic surfactant vesicles were synthesized as vanillin carriers: neutral niosomes formed by Span60 and cholesterol, positive charged niosomes formulated with cetyltrimethylammonium bromide (CTAB), and negatively charged niosomes formulated with sodium dodecyl sulfate (SDS). Niosomes synthesis was carried out with two commonly used methods: thin film hydration (TFH) and ethanol injection method (EIM). The niosomes synthesized were used to prepare two different materials: (i) a powder containing the lyophilized noisome with vanillin systems and (ii) a gelatin matrix film containing niosomes with vanillin. Lyophilization was carried out using maltodextrin as a cryoprotectant. The lyophilization of colloidal structures allows for storage at room temperature for long periods of time, keeping their organoleptic characteristics invariable. Niosomes were characterized before and after the lyophilization process in terms of morphological characterization, size, polydispersity index (PDI), and zeta potential. Moreover, niosomes cargo was evaluated by calculating the encapsulation efficiency (EE) and loading capacity (LC). Results showed that the use of the TFH method allowed us to obtain niosomes of 255 nm with high EE (up to 40%) and LC values higher than EIM. The lyophilization process decreased the LC of the vesicles prepared, but this decrease was mitigated by up to 20% when ionic surfactants were used on the membrane bilayer. Gelatin films are biodegradable materials suitable for food packing applications. The incorporation of a natural compound with antimicrobial activity would be a clear advantage for such an application. The films prepared were characterized in terms of morphology, water solubility, color, and transparency. Niosomes synthesized by thin film hydration had better chemical and physical properties to load vanillin. Especially in the case of application in films, niosomes with a negative charge, formed by SDS, and vanillin loaded gave better mechanical and chemical characteristics to the film.

## 1. Introduction

Vanillin is a chemical compound that can be extracted from the *Vanillin planifolia* plant. It is present at 1–2% in vanilla beans and is responsible for their characteristic flavor and smell [1]. It is widely used in the food and cosmetic sectors, but in recent times it has stimulated a keen interest in the pharmaceutical field. In addition to the improvement of the taste of pharmaceuticals, it has several bioactive properties: anti-carcinogenic, neuroprotective, antioxidant and antibiotics potentiation, and anti-quorum sensing activity in biofilms [2,3,4].

Antimicrobial resistance (AMR) is a global health problem since bacteria can create defense mechanisms that make the use of common antibiotics inefficient. Among these mechanisms are specific efflux pumps in the bacterial cell that expel the antibiotics. Many therapies targeting efflux pumps have been developed. Recent studies have confirmed the ability of Phyto-substances, including vanillin, to modulate the gens of efflux pumps and enhance the activity of antibiotics [3]. For example, vanillin extract inhibits the activity of efflux pumps in *P. aureginosa,* and it also helps restore the activity of traditional antibiotics like cephalosporines or levofloxacin in different strains of bacteria such as *S. aureus*, *E. coli*, and *P. aureginosa* [4,5].

Bacteria can grow on different surfaces in planktonic cells or create a film surrounded by a complex and resistant polysaccharide matrix known as a biofilm. In biofilms, cells communicate with each other using a system of transcriptional regulation of genes to activate or shut down metabolic pathways or cellular processes called quorum sensing. Vanillin has been found to modulate and inhibit part of the quorum-sensing signal cascade, and several studies are underway to confirm its antibiofilm activity [6].

Despite its interesting properties, vanillin has high hydrophilicity and low bioavailability, which limits its pharmacodynamics. To overcome the above, nanomaterials could play a useful role. They also offer additional features, such as increasing the stability of the molecule and controlling its release [7].

Nanovesicles are nanomaterials used as carriers for hydrophilic and hydrophobic compounds due to their structure consisting of a membrane surrounding an aqueous core. Niosomes are vesicles composed of non-ionic surfactants, cholesterol, and charged molecules. Common non-ionic surfactants used as membrane components are sorbitans (Spans), polysorbates (Tweens), and alkyloxyethylenes. The formulation is often governed by three parameters: hydrophilic-lipophilic balance (HLB) value, critical packaging parameter (CPP), and gel-liquid transition temperature (Tc) [8]. HLB value is an important parameter to determine the suitability of a surfactant to form vesicles, being values 3–6 the more appropriate. In this work, Span60 was used and had an HLB value of 4.7. It is capable of forming stable niosomes with the addition of small amounts of ionic surfactants or cholesterol, widely used to increase membrane robustness and fluidity [9]. Furthermore, cholesterol reduces the leakage of the encapsulated molecules in the vesicles [10].

The stability of the vesicles can be enhanced by the addition of charged molecules, increasing the surface charge density, which prevents clumping and fusion of the vesicles. They can also affect vesicle size distribution and the encapsulation efficiency (EE) of a biomolecule or drug [11]. In this study, two different charged surfactants were used for niosomes formulation: a positively charged molecule, cetyltrimethylammonium bromide (CTAB), and a negatively charged molecule, sodium dodecyl sulfate (SDS).

CTAB is also known for having antimicrobial properties and being commonly used in the pharmaceutical, cosmetic, and food fields. Above the critical micelle concentration (CMC), it forms micelles that act as encapsulating agents [12].

On the other hand, SDS improves vesicle stability influencing the zeta potential. In addition, it promotes the transdermal release of niosomes that encapsulate drugs suitable for this pharmacological route [13].

Mal et al. [12] demonstrated the antibacterial activity of these two components against *E. coli* and *B. subtilis*, used individually and as components in vesicles.

In this work, three different vesicle formulations were compared using two synthesis methods: ethanol injection method (EIM) and thin film hydration (TFH), considering the vesicle size and EE. Both methods have advantages and limitations. EIM is simple and reproducible and allows the synthesis of vesicles with a greater entrapment efficiency. On the other hand, it is difficult to remove all the ethanol during the synthesis, and the presence of ethanol causes the inactivation of some drugs. The TFH process creates vesicles with different layers that can be used for vesicle membrane compounds (lipids or surfactants). However, the process is less reproducible, time-consuming, and produces a non-uniform size distribution of vesicles [14,15,16].

In the second part of this work, once the most advantageous synthesis method in terms of encapsulation efficiency, morphology, and drug loading was selected, the focus was set on the stability and preservation of the vesicles in two applications.

The first one was the preparation of a powder by lyophilization. It is the most used technique to produce powder systems because it improves the stability of biopharmaceutical compounds, allows easier handling and storage, and the reduction of transport costs [17]. A cryoprotectant is always necessary to maintain the structural integrity of the vesicles. Without its protective effect, vesicles could be destroyed during the lyophilization process yielding drug leakage. Maltodextrin is one of the most commonly used polysaccharides used for this purpose [18]. The fresh suspensions were compared with the ones prepared after rehydration of the lyophilized powders in terms of shape, size, EE, and drug loading capacity (LC).

For the second application, the synthesized vesicles were incorporated into gelatin films for food packing purposes. Gelatin, a natural biopolymer, is increasingly being used in the form of films to protect food against oxidation and microorganisms attack, allowing long-term preservation. Gelatin is a biodegradable, biocompatible protein easy to procure. However, its high hydrophilicity and swelling result in practical difficulties while creating and using the films [19]. Therefore, those films require the addition of compounds that improve their physic-chemical and mechanical properties [20,21]. In the present study, the incorporation of vesicles with and without vanillin into gelatin films was carried out, and the resulting films were physically characterized.

## 2. Materials

Surfactants used for the membrane components were Span^®^ 60 (Sigma-Aldrich, San Luis, MI, USA), cholesterol stabilized at 96% (Acros Organics, Branchburg, NJ, USA), cetyltrimethylammonium bromide (CTAB) (Sigma-Aldrich), and sodium dodecyl sulfate (SDS) (Panreac, Barcelona, Spain). Surfactants were dissolved in absolute ethanol (J.T. Baker, Avantor, Allentown, PA, USA). For the aqueous phase, phosphate buffer saline (PBS) 10 mM, pH 7.4 was prepared using phosphate buffered saline tablets according to the manufacturer’s (Oxoid, Hampshire, UK) instructions. Vanillin at 99% was purchased from Sigma-Aldrich (MW = 152 g/mol). Maltodextrin (lot no.219425, MD, Pral, Barcelona) was added as a cryoprotectant. Gelatine films were prepared using gelatine from porcine skin (Sigma-Aldrich ref. G1890) and glycerol (Sigma-Aldrich 99.5% ref. G7893). The *E. coli* strain used for the antimicrobial activity test was kindly given by the Dairy Research Institute of Asturias (Instituto de Productos Lácteos de Asturias, IPLA-CSIC), Asturias, Spain.

## 3. Methods

### 3.1. Preparation of Nanovesicles

Vesicles were synthesized using two common methods: TFH and EIM. While the first method promotes the formation of multilamellar vesicles (MLV), the second one supports the formation of unilamellar vesicles (ULV) [22].

In both methods, an organic phase and an aqueous phase were prepared. A 50 g/L solution of surfactants was prepared in absolute ethanol and used as the organic phase. Two aqueous phases were used: ultra-pure (MilliQ, MQ) water and PBS. Since vanillin is a highly hydrophilic compound, it was added to the aqueous phase at a concentration of 0.2 M.

For the TFH method, 20 mL of the organic phase was poured into a flask. The removal of the solvent was performed under vacuum by a rotary evaporator (Buchi Labortechnik AG, Flawil, Switzerland) submerged in a thermostatic water bath at 45 °C, using 150 rpm of rotation speed and 70 mbar, allowing slow evaporation of the ethanol until a uniform formation of a transparent film around the flask took place. The second step was hydrating the film with the aqueous solution of 0.2 mM of vanillin. The formation of vesicles was facilitated by the rotation at 150 rpm while keeping a bath temperature of 60 °C for 2 h in the rotary evaporator. The vesicles size were then homogenized with a sonicator (Branson Ultrasonics Sonifier SFX150, Tamaulipas, Mexico) for 15 min using an amplitude of 55%, 500 W power, and 20 kHz frequency in continuous mode [23].

For the EIM method, the aqueous phase (50 mL) was stirred at 500 rpm (IKA ^®^, RCT standard) and heated until 60 °C while the organic phase (20 mL) was added with a syringe pump (KD Scientific ^®^) at a flow rate of 120 mL/h. Once the vesicles were formed, the ethanol was removed from the suspension using a rotary evaporator operating at 60 rpm, 40 °C, and 60–70 mbar until the volume was reduced to approximately 20 mL, ensuring complete ethanol evaporation. The vesicles were then homogenized by sonication, as described above.

**Figure 1:**
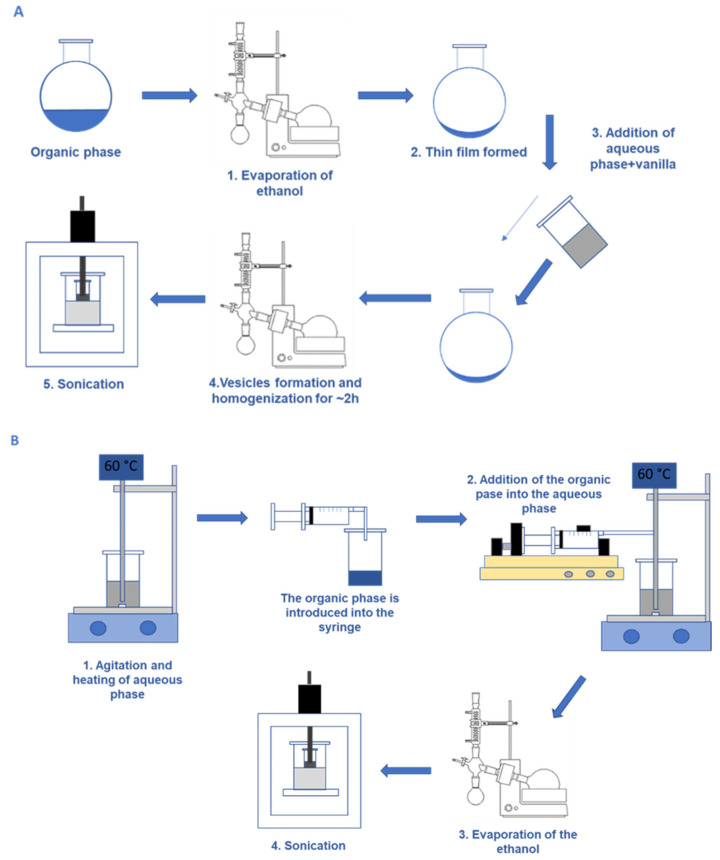
Schematic drawing of thin film hydration method (**A**) and ethanol injection (**B**) procedure used in the preparation of vesicles.

A schematic diagram of both preparation methods is presented in Figure 1.

The formulations tested were neutral, positive charged, and negative charged non-ionic vesicles and are summarized in Table 1.

### 3.2. Nanovesicles Purification

Vesicles were purified from possible contaminants, from the excess surfactants that do not form the membrane, and from the vanillin molecules that were not encapsulated. For that purpose, ultracentrifugation using ultra 0.5 mL centrifugal filter devices in a micro-centrifuge (Espresso Centrifuge, Thermo Electron Corp., Beverly, MA, USA) for 15 min at a 14,500 rpm. The supernatant was filtered using PES 0.22 µm pore diameter syringe filters prior to its analysis by RP-HPLC.

### 3.3. Lyophilization

After the synthesis of the nanovesicles, maltodextrin at a concentration of 20% (*v*/*v*) was added while applying a gentle stirring. The suspensions were then stored at −80 °C for 24 h. Then, the lyophilization process was performed under vacuum conditions (0.1 mbar) using a Telstar Cryodos Lyophilizer (Terrassa, Spain). The obtained powders were afterward resuspended in their corresponding aqueous phases, and their morphology and EE were assessed.

### 3.4. Film Preparation

Gelatin films were prepared by direct mixing 1 g of gelatin from porcine skin along with 0.2 g of glycerol in 10 mL of the vesicle suspension. The mixtures were then placed in a water bath at 60 °C for 25–30 min to dissolve the gelatin. Once dissolved, the solutions were poured into Petri dishes and allowed to dry in an oven at 40 °C for 24 h. Finally, the films were peeled with tweezers from the dishes. Gelatin films with empty vesicles and with vanillin-encapsulated vesicles were prepared.

### 3.5. Nanovesicles Characterization

Size distribution, average size, PDI, and zeta-potential were determined through dynamic light scattering (DLS) on a Zetasizer NanoZS series (Malvern Instruments Ltd., Malvern, UK).

The morphology of each formulation was studied through negative staining transmission electron microscopy (NS-TEM) using a JEOL-2000 Ex II Transmission Electron Microscope (Tokyo, Japan). The samples were placed on grids covered with carbon foil and stained with an aqueous solution of phosphotungstic acid (2% *w*/*v*) to obtain negative contrast and a correct visualization.

For the EE determination, the purified vesicles were mixed with methanol at a 1:10 ratio to break the structure of the vesicles and release the loaded drug. The amount of vanillin encapsulated was obtained by analyzing the samples with RP-HPLC (HP series 1100 chromatograph, Hewlett Packard, Agilent Technologies), with a Zorbax Eclipse Plus C18 column (4.6 mm × 150 mm, 5 μm, Agilent Technologies, Santa Clara, California, USA). Ultra-pure water (A) and methanol (B) were used as mobile phases in a linear gradient. The gradient started with 20% of B, obtaining 100% of B at 5 min and keeping it constant for 10 min. The flow rate was 0.8 mL/min. The vanillin peak considered was at λ = 280 nm.

EE values were calculated as the concentration ratio:

(1) $$\mathrm{EE}\left(\%\right)=\frac{\left(\;\mathrm{Concentration}\;\mathrm{of}\;\mathrm{vanillin}\;\mathrm{in}\;\mathrm{purified}\;\mathrm{vesicles}\right)}{\left(\mathrm{Concentration}\;\mathrm{of}\;\mathrm{vanillin}\;\mathrm{in}\;\mathrm{vesicles}\;\mathrm{before}\;\mathrm{purification}\right)}\times 100$$

The amount of vanillin inside the vesicles was expressed as loading capacity and calculated as follows:

(2) $$\mathrm{LC}\left(\%\right)=\frac{\mathrm{Wb}}{\mathrm{WT}}\times 100$$

where Wb corresponds to the mass of the biocompound encapsulated and WT is the total mass of the sample (biocompound and vesicle membrane compounds).

### 3.6. Nanovesicles Antimicrobial Activity Test

#### 3.6.1. Time Kill Assay

Time-kill assay was performed in a 96-well microplate, in which bacteria were inoculated in the bottom. The OD values using a wavelength of 600 nm were obtained at 2 h, 4 h, 6 h, 8 h, 12 h, and 24 h using a microplate reader (Varioskan Flash, Thermo Scientific). The concentration of bacteria cells (cells/mL) was calculated based on the correlation between the value of OD and the number of bacteria cells in mL [24].

*E. coli* was grown overnight in a liquid medium (Luria Bertani, LB) at 37 °C by transferring a single colony from a nutrient agar plate into 2 mL of LB. The concentration of the bacteria was measured by OD600, and then an aliquot of this growth medium was diluted to 1 × 10^5^ CFU/mL in LB. Afterward, a volume of 100 µL of this concentration of bacteria was inoculated into each well of a sterile 96-well plate containing 100 µL of free vanillin or encapsulated vanillin in the three vesicle formulations and empty vesicles. Positive control with only bacteria and negative control with only medium were also carried out. Experiments were performed in triplicate.

#### 3.6.2. Agar Well Diffusion Method

The antimicrobial activity of the vesicles was tested in vitro on *E. coli* by the agar well diffusion method evaluating the inhibition zones. Bacteria cultures (100 µL) were inoculated in about 15 mL of nutrient agar medium at 37 °C, poured into Petri dishes, and left to solidify. Holes were created in these cultures with a sterile glass tube with a diameter of 1 cm, and subsequently, 100 µL of the vesicle suspensions were transferred into each hole in the plate. One of the holes contained 100 µL of PBS as a control. The plates were incubated at 37 °C for 24 h. The formation of clear halos around the wells of the inoculated plates indicated the antimicrobial activity of the formulations tested. The diameter of the inhibitory zone of each halo was measured in cm for samples with and without vanillin encapsulated in vesicles. The diameter was expressed as the average of the triplicates.

### 3.7. Films Characterization

#### 3.7.1. Mechanical Properties and Thickness

Puncture strength (*PS*) and puncture deformation (*PD*) of the gelatin films were measured using a Texture Analyser TA.XT.plus (Stable Microsystems, Surrey, UK). Films were cut into squares of around 2 × 2 cm, placed on the test platform, and fixed with a cover plate. Samples were punctured at room temperature using a P/5 S probe (5 mm diameter) at a test speed of 1 mm/s. *PS* and *PD* were calculated according as follows [25]:

(3) $$PS=\frac{Fm}{Th}$$

(4) $$PD=\frac{\left(\sqrt{\left(D^{2}+R^{2}\right)}-R\right)}{R}$$

where *Fm* is the maximum force applied before the rupture of the film, *Th* is the film thickness (mm), *D* is the distance covered by the probe while it is in contact with the film until the film is broken (mm), and *R* is the radius of the hole in the plates (mm).

The thickness of the films was measured using a digital micrometer (Mitutoyo, Kawasaki, Japan). A point in the middle of the film was taken as a reference, and from these, four points were considered, with four different orientations, 2 cm away from the central one, for a total of 5 measured points. The reported film thicknesses are the average of the measured data.

#### 3.7.2. Color

The color of the films was measured through Lovibond^®^ LC100 Spectro colorimeter (Tintometer^®^ Group, Lovibond house, Amesbury, UK). Three parameters were considered: L* (lightness/brightness), a* (redness/greenness), and b* (yellowness). The whiteness index (*WI*) is correlated to these parameters according to the following expression:

(5) $$WI=100-{\left[{\left(100-L\right)}^{2}+a^{2}+b^{2}\right]}^{0.5}$$

#### 3.7.3. Optical Transmittance and Transparency Index

Light transmittance and transparency of the films were detected with a Helios gamma spectrophotometer (Thermo Fischer Scientific, Waltham, MA, USA). Films were cut and tested considering wavelengths from 200 to 800 nm. Transparency was calculated as follows [26]:

(6) $$\mathrm{Transparency}=\frac{A_{600}}{x}$$

where *A*_600_ is the film absorbance at 600 nm and *x* is the thickness of the film (mm).

#### 3.7.4. Water Solubility

The tested films were cut into strips about 2 × 4 cm and immersed in 20 mL of a pH 7.0 aqueous solution containing 10% (*w*/*w*) 1M Trizma^®^ hydrochloride solution pH 7.0 (Sigma-Aldrich). The immersed film was kept at room temperature for 24 h. Afterward, the film fragments were recovered by filtration using Whatman N°1 paper and dried in an oven at 105 °C for 24 h. At the same time, a set of identical film sheets was dried under the same conditions without first dissolving.

Water solubility (*WS*) was calculated as:

(7) $$WS\left(\%\right)=\frac{\left(m_{1}-m_{2}\right)}{m_{1}}\times 100$$

where *m*_1_ is the mass in grams of the dry films, and *m*_2_ is the mass in grams of the solubilized films.

### 3.8. Statistical Analysis of Data

All experiments were performed in triplicate. Results are shown as arithmetic mean values. To analyze differences between test groups, an analysis of variance (ANOVA) was performed. Significant differences between groups were determined using Fischer’s least significant difference (LSD) test. Levels of *p* < 0.05 were considered significant. Analysis was performed with IBM^®^ SPSS^®^ Statistics 25 statistical software.

## 4. Results and Discussion

### 4.1. Vesicles Preparation and Characterization

The nanovesicles prepared using the three formulations studied were first synthesized without any compound encapsulated by the two aforementioned methods: TFH and EIM.

#### 4.1.1. Physical Characterization of Vesicles

**Figure 2:**
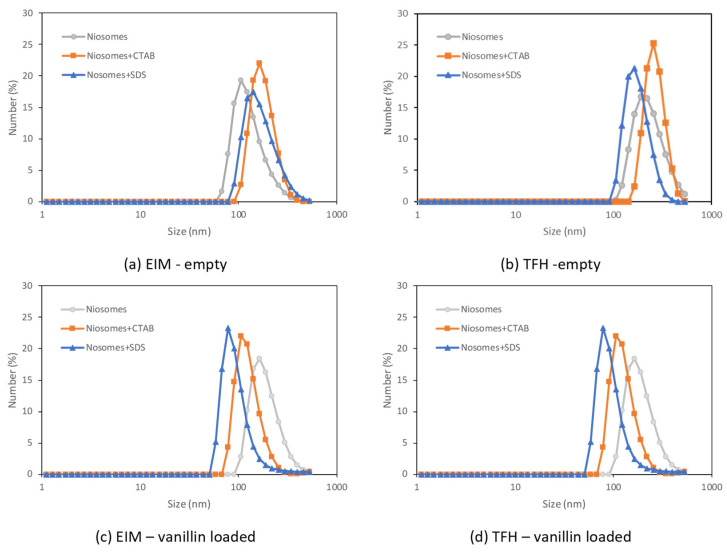
Particle size distribution of the three systems (niosomes, niosomes + CTAB, and niosomes + SDS) prepared by (**a**,**c**) ethanol injection (EIM) and (**b**,**d**) thin film hydration (TFH) without (**a**,**b**) and with (**c**,**d**) vanillin encapsulated.

Nanovesicles were characterized in terms of mean particle size, particle size distribution, morphology, zeta potential, and EE to study the effect of the formulation selected and the method used for nanovesicle preparation. Results are shown in Figure 2 and Table 2.

The use of EIM and TFH methods led to different particle distributions for the three formulations tested. Previously published studies have concluded that EIM produces smaller niosomes than TFH [27,28]. However, the type of surfactant used affects vesicle size. Span 60 with an HLB of 4.7 forms generally vesicles with small size. An explanation for this can be that with a decrease in hydrophilic groups, the surface energy of niosomes decreases, which provokes a decrease in size [29].

The presence of vanillin increased the particle size of all vesicles formulated by the two methods used, in agreement with previous works [30,31]. The use of the TFH method for the preparation of vanillin-loaded vesicles also presented a large dependence on size with the vesicle membrane formulation. Smaller vesicles were always obtained when ionic surfactants (SDS or CTAB) were used in their formulation. However, vanillin seems to have less influence on the size of the vesicles prepared with an anionic surfactant. This can be explained by a denser packing of the SDS molecules in the vesicle membrane.

The presence of surfactants with high HLB values, such as SDS and CTAB, produced a slight increase in the particle size obtained when the EIM method was used. This could be associated with an increase in the curvature radius during the self-assembling that occurs when those surfactants get incorporated into the vesicle membrane bilayer. On the other hand, in the TFH method, the presence of surfactants with high HLB value produced a high reduction (larger than 50%) in particle size. This indicates a denser packing of the molecules during the hydration step, producing multilamellar vesicles. This behavior has been reported before [32]. Moreover, larger standard deviations were observed when nanovesicles were produced by the TFH method in comparison to EIM. This reduced reproducibility was also expected.

Nanovesicles prepared with the TFH method presented the largest PDI values, that varied from 0.17 to 0.54, indicating more polydispersity of the systems. The values registered for EIM ranged between 0.11 and 0.07. The large values obtained for TFH indicate the instability of the systems due to the fusion of smaller vesicles promoted by surface interactions [28,33].

The physical stability of the vesicles can be assessed by the surface charge measured through the zeta potential [34]. The ionic zeta potential of particles in a colloidal system depends on the type and concentration of surfactants and active and stabilizing material. The surface charge is key for the vesicle to interact with the molecule to be encapsulated and with the cell membrane. Typically, low zeta potential is obtained when non-ionic or neutral surfactants are used [35].

Niosomes prepared without ionic surfactant on the vesicle membrane layer presented slightly negative surface charges. The introduction of a cationic surfactant (CTAB) on the membrane vesicle layer produced a positive charge, and the use of an anionic surfactant (SDS) produced a negative charge on the particle surface without large differences between both preparation methods used. SDS had been used in previously published studies to increase the surface charge of the niosomes and improve the stability of vesicles. Vesicles produced by the TFH method showed larger absolute zeta potential than those produced by EIM, indicating higher electrostatic stability of the nanovesicles.

The loaded molecule can sometimes influence the zeta potential [36], but in this study, the presence of vanillin did not seem to influence the zeta potential. Values in both cases (for empty and loaded vesicles) were around −40 mV for formulations containing the SDS anionic surfactant, 35 mV for those containing the CTAB cationic surfactant, and close to −12 mV for the ones prepared with non-ionic surfactants.

As a general trend, higher EE was registered when TFH was used as a loading method. Recent studies describe that TFH is one of the methods that offer the best EE values even for hydrophilic molecules that are typically encapsulated in the aqueous cavity located inside the vesicle [33]. This can be explained by considering that when multilamellar vesicle-type vesicles are prepared, greater resistance to the permeation of the encapsulated molecule into the external aqueous phase arises. It was also reported that using TFH as a synthesis method, the drug encapsulation increases to 60–75%. The use of THF is especially interesting when hydrophobic drugs or low aqueous solubility are encapsulated. These hydrophobic molecules are located at the vesicle membrane layer [10,37].

It is also important to point out that vanillin presents a low molecular weight (MW = 152 g/mol). Low MW compounds like pilocarpine (MW = 208.26 g/mol) were found to be more difficult to encapsulate and also showed undesired release during storage [28].

The presence of ionic surfactants as co-stabilizers in the formulation resulted in lower EE values. Nevertheless, for both methods, niosomes with CTAB had higher EE than those prepared with SDS. This could be linked to a possible interaction between vanillin and the CTAB surfactant. CTAB produces a favorable environment for the deprotonation of the vanillin molecule. The electrostatic attraction between the negatively charged vanillin molecule and the positively charged CTAB molecule was demonstrated in previous studies. It was shown to promote the trapping of vanillin into CTAB micelles [38].

**Figure 3:**
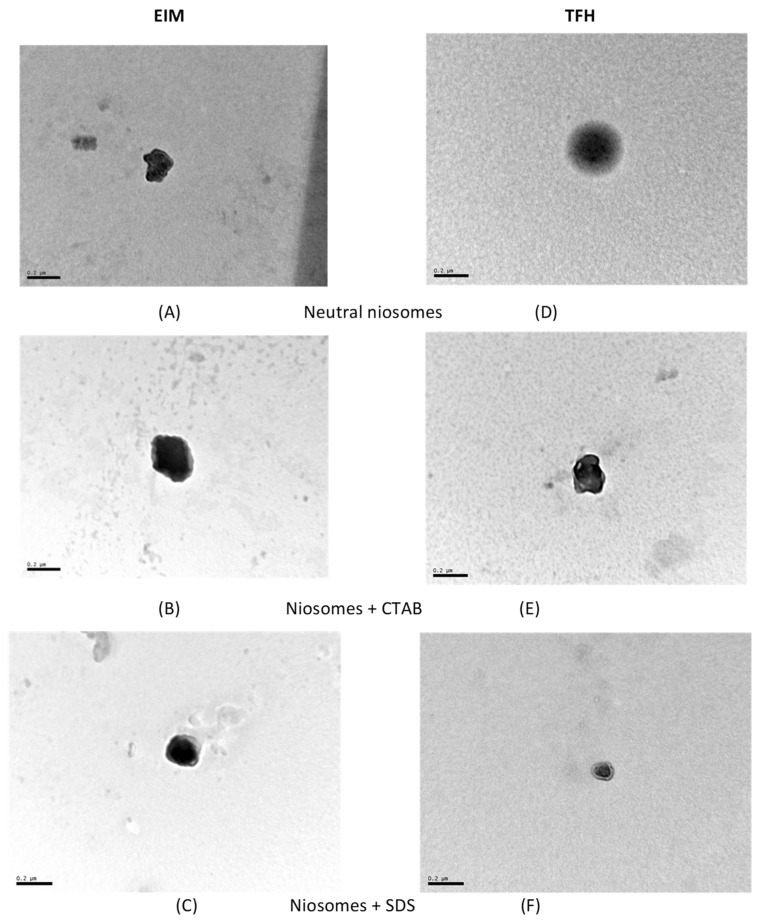
TEM images of vesicles prepared by ethanol injection (EIM) (**A**–**C**) and thin film (TFM) (**D**–**F**). Formulations: (**A**,**D**) niosomes, (**B**,**E**) niosomes with CTAB, and (**C**,**F**) niosomes with SDS.

The morphology of the vesicles was analyzed using TEM, and the results are shown in Figure 3. The sizes were found to corroborate the values obtained by DLS.

#### 4.1.2. Antimicrobial Activity of Nanovesicles Loading Vanillin

**Figure 4:**
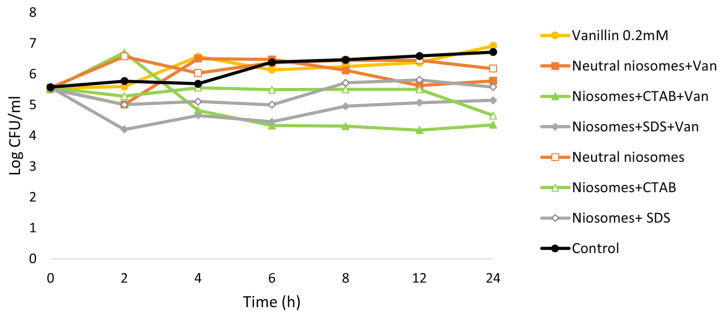
Time kill curves of each formulation of niosomes with and without vanillin compared to the control and free vanillin.

The nanovesicles prepared by THF, which showed the best EE values, were next tested for their antimicrobial activity. Time kill assay and agar well diffusion method were carried out. Figure 4 shows the effect on bacterial growth after exposure to these materials for 24 h.

Both negatively charged niosomes and niosomes with positive charge loaded with vanillin showed a bactericidal effect, whereas non-encapsulated vanilla did not have a significant effect on microbial growth. Empty niosomes + SDS and niosomes + CTAB exhibited a bacteriostatic effect, as the concentration of bacteria remained similar over time. However, no significant effect of neutral vesicles was observed compared to the growth control. Only after 24 h of exposure to neutral niosomes loaded with vanillin, a decrease in the bacterial density could be observed.

**Figure 5:**
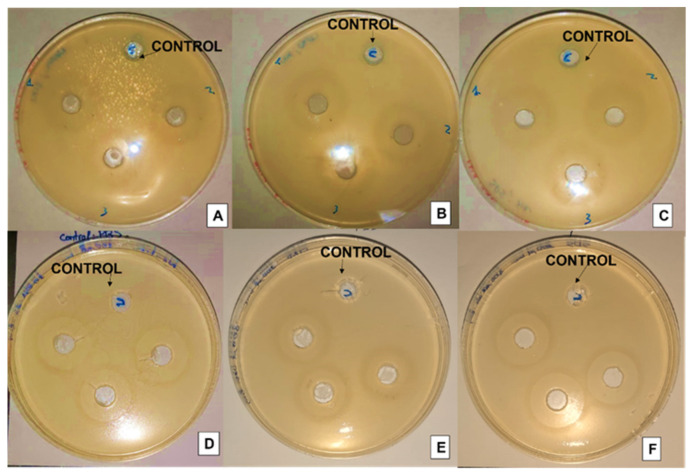
(**A**) Niosomes loaded with vanillin, (**B**) Niosomes with CTAB loaded with vanillin, (**C**) Niosomes with SDS loaded with vanillin, (**D**) Niosomes without vanillin, (**E**) Niosomes with CTAB without vanillin, (**F**) Niosomes with SDS without vanillin.

The activity of each formulation with and without vanillin encapsulated on *E. coli* was compared. The observations are shown in Figure 5 and in Table 3.

The diameter of each halo was measured: the longest halo was the one produced by niosomes + SDS giving a value of 1.42 cm, followed by the neutral niosomes with 1.33 cm, and the smaller halo was obtained for niosomes + CTAB (1.03 cm). This allows us to conclude that the maximum inhibition was obtained with loaded negatively charged vesicles. A similar tendency was observed in other studies that demonstrated that micelles formed by SDS produced larger halo against bacteria than CTAB micelles [12]. The halo of the non-loaded vesicles was also measured as blank. It was possible to see a different behavior in its antimicrobial activity. In neutral niosomes and niosomes with SDS, the diameter is reduced when vanillin was not present in the formulation. For niosomes with CTAB similar value was obtained with and without vanillin loaded. It is known that CTAB is a surfactant with antimicrobial and bactericidal properties. For this reason, it is presumable, though, that the inhibition observed for niosomes + CTAB is mostly due to the presence of the CTAB surfactant.

### 4.2. Vesicles Lyophilization

**Figure 6:**
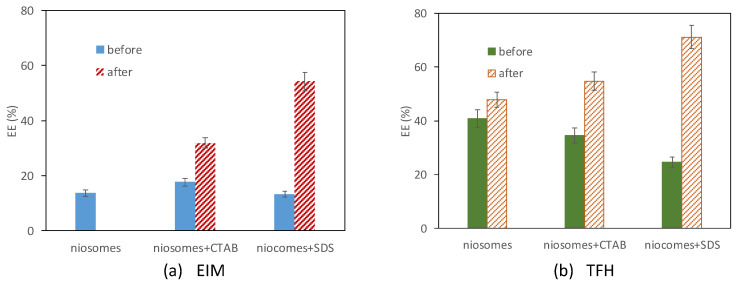
Encapsulation efficiency of vesicles before and after the lyophilization process, (**a**) prepared by ethanol injection method and (**b**) prepare by thin fim hydration method.

Vesicles loaded with vanillin prepared by both methods, EIM and THF, were frozen at −80 °C and lyophilized. After lyophilization, they were restored with MQ water. EE of the system before and after the lyophilization process was measured and compared. Values are presented in Figure 6.

It was observed that after lyophilization, the EE of the nanovesicles increased in most cases for all formulations and both preparation methods used (Figure 6). The exception is the neutral niosomes, in which encapsulated vanillin was not observed after the lyophilization process when they were prepared by EIM (Figure 6a). It is important to point out the increase of EE after the lyophilization process when ionic surfactants were used, especially the anionic surfactant SDS for both preparation methods used.

In this type of process is also important to quantify the LC of the systems since the preparation process could produce a partial loss of the biocompound. The LC was calculated according to Equation (2), in which the mass of the vanillin and the mass of the vesicle membrane compounds are considered. Two different values were obtained for each vesicular system: (i) total, which refers to the total mass of the membrane compounds and the total vanillin present in the sample, and (ii) encapsulated, which relates to the total mass of the membrane compounds and the encapsulated vanillin in the vesicles (after vesicle purification).

To determine the LC values of the vesicles before and after lyophilization, a calibration curve of absorbance versus vanillin concentration was performed (Figure S1 of Supplementary Material).

**Figure 7:**
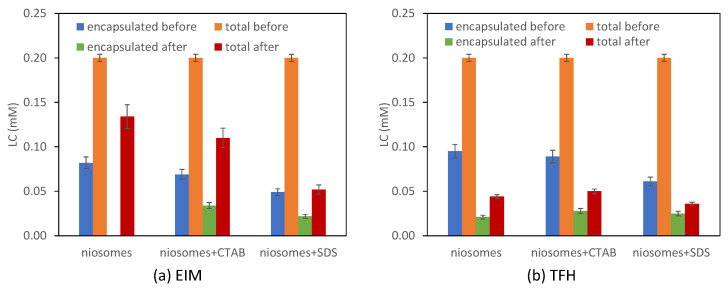
Loading capacity of Vanillin, total and encapsulated, before and after lyophilization process for (**a**) vesicles prepared by EIM method and (**b**) vesicles prepared by TFH method.

The calibration curve was used to calculate the vanillin concentration of the systems. The calculated LC values are summarized in Figure 7.

The preparation method used to produce the vesicles seems not to have a large influence on the LC values obtained for the same formulations. However, comparing the values of the encapsulated concentration before and after the lyophilization process, the LC is reduced after the lyophilization process, up to 50% of value when vesicles prepared by EIM are treated and even up to 70% when vesicles prepared by TFH were used. As previously reported, lyophilization provokes several stresses and instability, and aggregation to the vesicles during the freezing and drying lyophilization steps [39].

The reduction observed on the LC is more than 20% larger when neutral niosomes were tested, which is especially noticeable when the EIM method was used, in which case any encapsulated vanillin was observed after the lyophilization process. For systems in which CTAB and SDS are present as membrane components, encapsulated LC decreases especially. It must be considered that the CTAB, in the presence of organic solvents such as methanol, is used to break the membrane bilayer of the vesicles and liberates the encapsulated molecule, interrupts the micellization process probably due to the entry of the solvent inside the micelles [40]. Therefore, the results obtained will not only be influenced by lyophilization, which involves a loss of load during the process but also using surfactants chosen for nanovesicles formulation.

### 4.3. Films Preparation and Characterization

As mentioned before, nanovesicles synthesized by the THF method showed better results in terms of size, EE, and LC. For these reasons, these were chosen for their incorporation in gelatin biodegradable films. Films with vanillin-loaded and non-loaded vesicles were prepared, characterized, and compared.

#### 4.3.1. Mechanical Properties and Thickness

The thickness of the produced films is summarized in Table 4 with the measures of the PS and PD. The films have a similar thickness value, and it can be noted that the presence of vanillin did not have an important influence. As far as the PS and PD are concerned, there were no significant differences between them. Only in the case of the film with niosomes + SDS was it observed that PS and PD decreased by the presence of vanillin, giving values of 66% lower for PS and 44% for PD.

Observing the results obtained with films without vanillin, those prepared with niosomes + SDS showed slightly stronger properties than the ones with neutral niosomes and niosomes + CTAB. For instance, the PS was nearly 50% higher than that of the neutral niosomes film, and the PD was around 25% higher. These results can be considered that the addition of SDS, with a negative charge, enhanced gelatin film formation producing a denser film, thereby enhancing the PS and PD of the film produced.

#### 4.3.2. Color

**Figure 8:**
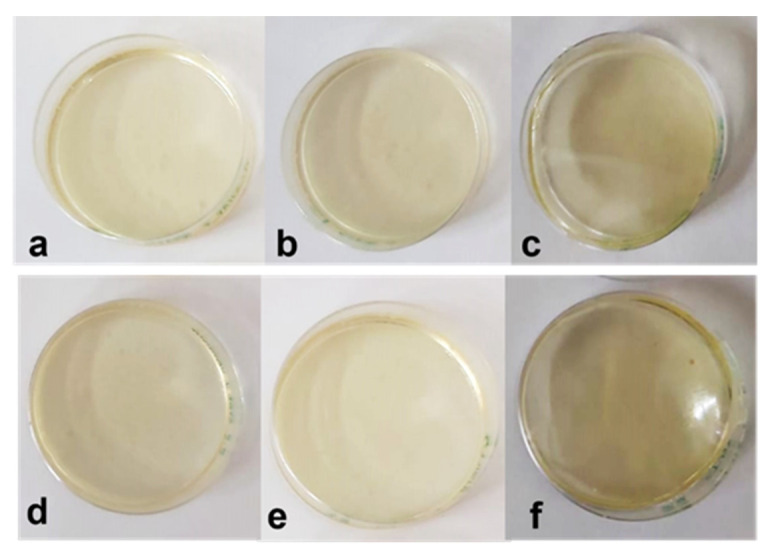
Images of the produced films, with non-loaded niosomes (**a**–**c**), with loaded niosomes (**d**–**f**), with neutral niosomes (**a**,**d**), with niosomes + CTAB (**b**,**e**), and with niosomes + SDS (**c**,**f**).

Films color is one of the properties to consider for food packaging applications. The films showed a yellowish color to the naked eye, and the presence of vanillin accentuated it more (Figure 8). This was confirmed by the calculation of WI, which showed a lower value for films with vanillin. WI measurements are resumed in Table 4.

The addition of vanillin reduced the a* value of the film and increased the b* and c* values. This means that, with no change in brightness, due to the addition of vanillin, the film color shifts toward green and toward yellow, while WI decreases. Measurements of L*, a*, B*, and c* values are summarized in Table S1 from Supplementary material.

#### 4.3.3. Optical Transmittance and Transparency Index

Light transmittance of films was analyzed with a wavelength range between 200 and 800 nm. For food packaging applications, it is important to consider a low transmission in the UV range from 200 to 400 nm to avoid food oxidation [41].

**Figure 9:**
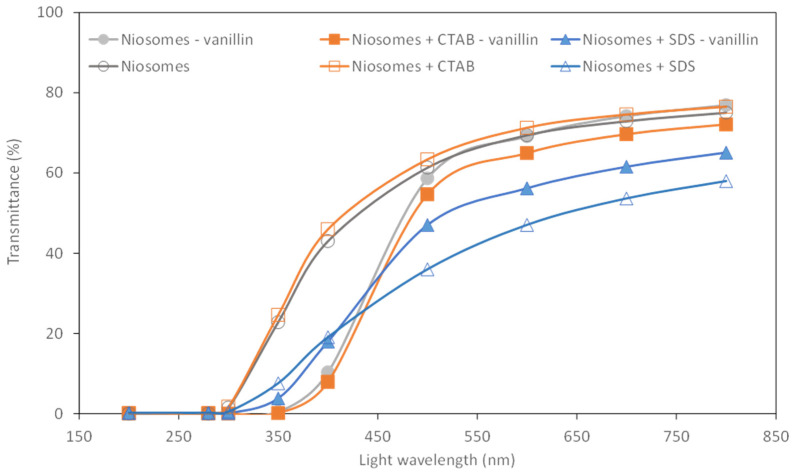
Light transmittance of films at different wavelengths.

Transmittance results for the characterized films are presented in Figure 9. In terms of light transmittance, it can be appreciated that films with vanillin-loaded niosomes presented more suitable transmittance values for food applications than those without vanillin. Low transmittance (less than 10–20%) was registered for wavelengths lower than 400 nm, giving the lower values for systems that contained neutral niosomes and niosomes + CTAB. At the same time, films containing non-loaded niosomes presented larger transmittance on the UV regime for the two systems mentioned, neutral niosomes and niosomes + CTAB.

For films prepared with niosomes + SDS, no large differences were observed with the presence or absence of vanillin, arising on the UV regime an intermediate behavior, presenting lower transmittance than the other two formulations without vanillin but larger than for the other two formulations with vanillin encapsulated.

Regarding the visible wavelength range, between 400–800 nm, those films produced with non-loaded and loaded-niosomes + SDS presented lower values. Non-loaded neutral niosomes, non-loaded niosomes + CTAB, and loaded neutral niosomes are the three systems with higher transmittance, which could be more satisfactory from the consumer perspective.

Table 4 also presents the transparency index measured at 600 nm, taking into account the film thickness. In this aspect, it can be observed that films with niosomes + SDS presented the larger transparency, especially when non-loaded niosomes + SDS were used.

#### 4.3.4. Water Solubility

The solubility of the films was calculated at pH 7 at room temperature. The films showed a solubility value of around 65%. However, the film with vanillin-loaded niosomes + SDS encapsulated dissolved completely in the water, as it is shown in Table 4.

This behavior indicated once again that the basic gelatin molecules that make up the film are synergized by SDS and vanillin, which affects the intermolecular connection, resulting in higher solubility and lower mechanical properties of the film.

## 5. Conclusions

Vanillin is a compound that has attracted a lot of attention in recent times in the research for new natural bio compounds with antimicrobial action. It is possible to exploit its advantageous properties and implement the activity of conventional antibiotics using nanoformulations and drug delivery as a tool. Vesicles are widely used in this field as drug and biocompound carriers. In the present work, two synthesis methods of nanovesicles were compared and evaluated to choose the most suitable for this bio compound. The Long-lasting and storage of the nanoformulations were analyzed through the lyophilization process in terms of the content of Loading Capacity (LC) and Encapsulation Efficiency (EE) of Vanillin.

Regarding the synthesis of empty niosomes, the TFH method turned out to be the most difficult to control with respect to size and, in addition, presented high polydispersity of the samples. On the other hand, this method presented the highest values of the zeta potential both for the niosomes formulated with CTAB and for those with SDS.

On the other hand, EIM is highly reproducible and has control over size. The samples presented great monodispersity due to their narrow size distributions.

The size of all formulated nanovesicles increased when vanillin encapsulation was carried out, being larger when the TFH method was used compared to the EIM method. Therefore, the importance of the formulation on the final nanovesicle size was observed, especially when the TFH method was used. In all cases, a decrease in size in the presence of SDS. However, in the case of niosomes + CTAB, this reduction was just noticeable when the TFH method was used. This highlights the importance of the proper selection of the preparation method depending on the formulation desired.

The EE obtained was higher for all the formulations prepared with the TFH method. Therefore, among those tested in this work, THF is the most appropriate preparation method for vanillin encapsulation. EE also increased in the presence of an ionic surfactant, either cationic or anionic, due to the co-stability that these provide to the vesicle structure, avoiding the undesired release of the molecules from the inner vesicle aqueous core.

The antimicrobial activity of vesicles in the presence and absence of encapsulated vanillin has been tested. Vanillin-loaded vesicles present higher antimicrobial activity, especially in cases where ionic and anionic surfactants are used.

The lyophilization process produced an increase in EE in all cases. This process presents opportunities to obtain high EE. However, the amount of Vanillin lost during the drying process was more than 50%, which is reduced (up to 20%) by using ionic surfactants on the membrane bilayer of the vesicle. An accurate selection of the cryoprotective agent nature and concentration used should be necessary to increase the LC of lyophilized nanovesicles loaded with hydrophilic molecules of low molecular weight.

It has been demonstrated that the presence of vanillin influenced the chemical and physical properties of the gelatin films. In fact, especially in films containing negatively charged vesicles, the PS and PD decreased considerably. As far as transmittance is concerned, the presence of vanillin positively influenced films by decreasing the transmittance index, in the ultraviolet light range, which enhances food preservation and avoids its oxidation. Even films with negatively charged vesicles showed a more advantageous behavior and presented high-water solubility at neutral pH. Further work should be ascertained to improve this last important feature of the film.

Among those tested, the optimal formulation for vanillin incorporation is the one using Span 60:Cholesterol:CTAB as vesicle membrane compounds. It offers high EE, high resistance during the lyophilization process, and suitability to be incorporated into a biodegradable film. The resulting material has appropriate physical properties to be used in food packaging processes. Nevertheless, to improve the antimicrobial activity, further experiments should be carried out with the aim of increasing the amount of encapsulated vanillin.

## Figures and Tables

**Table 1:** Composition of the different nanovesicles formulated using a total concentration of membrane components of 50 mM.

	Membrane Components	Molar Fraction
I	Span 60, Cholesterol	0.67; 0.33
II	Span 60, Cholesterol, CTAB	0.45; 0.22; 0.33
III	Span 60, Cholesterol, SDS	0.45; 0.22; 0.33

**Table 2:** Size, PDI, and zeta potential for the nanovesicles prepared with the three formulations by ethanol injection and thin film hydration.

	Formulation	Size (nm)		PDI		Z-Pot (mV)		EE (%)	
		EIM	TFH	EIM	TFH	EIM	TFH	EIM	TFH
Empty vesicles	Niosomes	106 ± 12	255 ± 42	0.11 ± 0.01	0.54 ± 0.23	−13 ± 3	−12 ± 7	-	-
	Niosomes + CTAB	164 ± 18	51 ± 35	0.07 ± 0.02	0.49 ± 0.06	28 ± 5	37 ± 6	-	-
	Niosomes + SDS	142 ± 13	79 ± 31	0.12 ±0.02	0.51 ± 0.26	−37 ± 4	−43 ± 7	-	-
Loaded vesicles	Niosomes	191 ± 28	319 ± 40	0.06 ± 0.02	0.17 ± 0.03	−11 ± 2	−12 ± 3	14 ± 3	41 ± 7
	Niosomes + CTAB	235 ± 32	257 ± 38	0.06 ± 0.05	0.22 ± 0.12	31 ± 4	41 ± 6	18 ± 4	35 ± 6
	Niosomes + SDS	157 ± 13	93 ± 32	0.07 ± 0.02	0.45 ± 0.16	−42 ± 3	−49 ± 3	13 ± 2	25 ± 5

**Table 3:** Diameter of inhibitory zones caused by empty niosomes and niosomes loaded with vanillin against *E. coli*.

Samples	Diameter (cm)	Samples	Diameter (cm)
Niosomes	0.96	Loaded niosomes	1.33
Niosomes + CTAB	1.00	Loaded niosomes + CTAB	1.03
Niosomes + SDS	1.03	Loaded niosomes + SDS	1.42

**Table 4:** Physical properties of each film with vesicles loaded vanillin and non-loaded niosomes.

	Films with	Thickness(mm)	PS (N/mm)	PD (%)	WI	Transparency Index	Solubility (%)
	Niosomes	1.1 ± 0.1 ^ab^	62 ± 10 ^b^	176 ± 3 ^b^	80 ± 5 ^ef^	0.18 ± 0.04 ^g^	68 ± 1 ^i^
Non-loaded vesicles	Niosomes + CTAB	1.07 ± 0.08 ^ab^	79 ± 7 ^bc^	187 ± 31 ^b^	82 ± 4 ^f^	0.16 ± 0.01 ^g^	65 ± 11 ^i^
	Niosomes + SDS	1.06 ± 0.09 ^ab^	90 ± 11 ^c^	237 ± 9 ^c^	78.8 ± 0.5 ^ef^	0.3 ± 0.1 ^h^	62 ± 4 ^i^
	Niosomes	1.17 ± 0.08 ^a^	68 ± 11 ^b^	175 ± 2 ^b^	72 ± 2 ^de^	0.17 ± 0.03 ^g^	65 ± 3 ^i^
Loaded vesicles	Niosomes + CTAB	1.1 ± 0.1 ^ab^	77 ± 14 ^bc^	177 ± 20 ^b^	68 ± 9 ^d^	0.17 ± 0.03 ^g^	67.5 ± 0.6 ^i^
	Niosomes + SDS	0.96 ± 0.07 ^b^	31 ± 9 ^a^	132 ± 22 ^a^	77.3 ± 0.8 ^ef^	0.27 ± 0.07 ^gh^	99 ± 8 ^k^

The same superscript letter indicates no significant differences between measurements.

## Data Availability

Not applicable.

## Supplementary Materials

The following supporting information can be downloaded at: https://www.mdpi.com/article/10.3390/membranes13010095/s1, Figure S1: Calibration curve of vanilla determination. Measured at λ = 28; Table S1: Colour of the films with loaded and non-loaded vesicles with vanilla.
