# Supplementary material for: Nanovesicles as Vanillin Carriers for Antimicrobial Applications
Source: Membranes (Basel). 2023 Jan 11;13(1):95. doi: 10.3390/membranes13010095 (PMC9865702; doi:10.3390/membranes13010095)
Supplement: Supplementary file 1 [file membranes-13-00095-s001.zip › membranes-2090059-supplementary.pdf]

Supplementary material

Nanovesicles as vanillin carriers for antimicrobial applications

*Verdiana Marchianò [1,2], Maria Matos[1,3], Miriam López[1], Shihan Weng[1], Esther Serrano-Pertierra[2,3], Susana Luque[1], M. Carmen Blanco-López[2,3], Gemma Gutiérrez[1,3]**

[1]Department of Chemical and Environmental Engineering, University of Oviedo, Julián Clavería 8, 33006 Oviedo, Spain

[2]Department of Physical and Analytical Chemistry, University of Oviedo, Julián Clavería 8, 33006 Oviedo, Spain

[3] Instituto Universitario de Biotecnología de Asturias, University of Oviedo, 33006, Spain

*Correspondence: gutierrezgemma@uniovi.es; Tel.: +34 985103510; Fax: +34 985103434

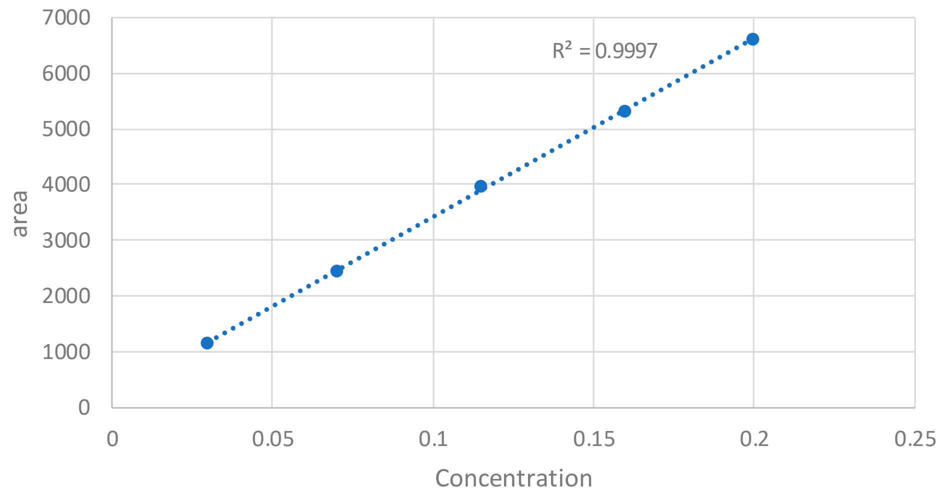

Figure S1. Calibration curve of vanilla determination. Measured at λ=280nm.

Table S1- Colour of the films with loaded and non-loaded vesicles with vanilla

	Films with:	L*	a*	b*	C*
Solubility (%)	Niosomes	81.100 ± 4.618^{a}	2.967 ± 0.153^{c}	5.000 ± 2.685^{a}	5.967 ± 2.223^{a}
Non-loaded vesicles	Niosomes+CTAB	82.967 ± 4.086^{a}	2.667 ± 0.116^{bc}	3.967 ± 2.376^{a}	4.867 ± 2.113^{a}
	Niosomes+SDS	80.900 ± 0.361^{a}	3.000 ± 0.000^{c}	8.700 ± 0.503^{ab}	9.200 ± 0.794^{ab}
	Niosomes	81.000 ± 2.152^{a}	-0.433 ± 0.306^{a}	21.133 ± 1.250^{cd}	21.167 ± 1.193^{cd}
Loaded vesicles	Niosomes+CTAB	78.167 ± 8.985^{a}	0.300 ± 1.559^{ab}	23.600 ± 3.175^{d}	23.633 ± 3.232^{d}
	Niosomes+SDS	83.167 ± 0.643^{a}	0.367 ± 0.945^{ab}	15.200 ± 1.540^{bc}	15.200 ± 1.540^{bc}

The same superscript letter indicates not significant differences between measurements
